# Hybrid brain–computer interface for biomedical cyber-physical system application using wireless embedded EEG systems

**DOI:** 10.1186/s12938-016-0303-x

**Published:** 2017-01-07

**Authors:** Rifai Chai, Ganesh R. Naik, Sai Ho Ling, Hung T. Nguyen

**Affiliations:** Centre for Health Technologies, Faculty of Engineering and Information Technology, University of Technology, Sydney, NSW 2007 Australia

**Keywords:** Brain–computer interface, Cyber physical system, Electroencephalography, Artificial neural network, Hybrid system, Embedded system

## Abstract

**Background:**

One of the key challenges of the biomedical cyber-physical system is to combine cognitive neuroscience with the integration of physical systems to assist people with disabilities. Electroencephalography (EEG) has been explored as a non-invasive method of providing assistive technology by using brain electrical signals.

**Methods:**

This paper presents a unique prototype of a hybrid brain computer interface (BCI) which senses a combination classification of mental task, steady state visual evoked potential (SSVEP) and eyes closed detection using only two EEG channels. In addition, a microcontroller based head-mounted battery-operated wireless EEG sensor combined with a separate embedded system is used to enhance portability, convenience and cost effectiveness. This experiment has been conducted with five healthy participants and five patients with tetraplegia.

**Results:**

Generally, the results show comparable classification accuracies between healthy subjects and tetraplegia patients. For the offline artificial neural network classification for the target group of patients with tetraplegia, the hybrid BCI system combines three mental tasks, three SSVEP frequencies and eyes closed, with average classification accuracy at 74% and average information transfer rate (ITR) of the system of 27 bits/min. For the real-time testing of the intentional signal on patients with tetraplegia, the average success rate of detection is 70% and the speed of detection varies from 2 to 4 s.

## Background

The new emerging frontier of technology modeling, cyber-physical systems (CPS), is related to the generation of new systems with the integration of the physical systems and real-time computational intelligence capabilities. This creates a fully intelligent system to tackle a variety of application fields such as robotics, intelligent buildings, biomedical and healthcare, energy, manufacturing control systems, and avionics [1, 2].

In biomedical CPS fields, especially in cognitive neuroscience, there has been significant interest in creating assistive technologies to improve the quality of life of individuals with disability in areas such as system integration with motor prosthesis, wheelchair control, environmental control and other applications [3–5]. One of the important challenges in CPS is human–machine interfacing. An integrated cyber-physical system should meet the real-time operational requirements including portability, reliability and predictability. A dedicated embedded system together with wireless technology would be an ideal platform for such an integrated real-time CPS [2, 6, 7].

In the application of wheelchair control for people with disabilities, various hands-free technologies have been used to replace the joystick including sip-and-puff [8], chin controller [9], muscle based system [10], voice recognition [11], tongue controller [12] and head movement systems [13]. These technologies have their own benefits and drawbacks. In practical situations, the user may feel uncomfortable with the operation of a sip-and-puff or a chin or tongue controller. Noisy environments can be problematic for voice recognition systems. Muscle and head movement technology are targeted for disabled individuals who are still able to provide the relevant body movement for real-time control. Certain neurological conditions such as amyotrophic lateral sclerosis (ALS), cervical spinal cord injury and brain stem stroke may lead to severe motor paralysis and mobility restriction referred to as a locked-in syndrome [14]. A brain computer interface (BCI) could be used as an alternative solution for these disabled individuals by converting brain activities to provide a means of control and communication [3, 5, 11, 15].

The acquisition techniques which are available in BCI systems can basically be classified into invasive and non-invasive brain measurements. The invasive methods include intra-cortical recording using microelectrodes and electrocorticography (ECoG). Although these methods could provide a better temporal resolution and quality, they have drawbacks such as the risk of infection and scarring post-surgery [16]. The electroencephalography (EEG) as a non-invasive approach has good temporal resolution, portability and low cost compared to other non-invasive BCI methods, such as functional near infrared spectroscopy (fNIRS) and functional magnetic response imaging (fMRI) [17, 18].

The applications of BCI–EEG as viewed from the aspect of mental strategies can be divided into either selective attention or spontaneous mental signal methods. The P300 [19, 20] technique and the steady state visual evoked potential (SSVEP) [4, 21] technique are examples of the selective attention method. For these the user needs to concentrate on external stimuli that flash in succession (P300) or continuously in a certain frequency (SSVEP). The BCI speller system is a good example of an application using selective attention. BCI systems relying on spontaneous mental signals generated voluntarily by the user may include self-regulation of the slow cortical potential (SCP) [22], control of the sensory motor rhythm (SMR) [23, 24] and the event-related desynchronization/synchronization (ERD/ERS) [25, 26] which focuses on the motor imagery area such as by imagining hand, foot or tongue movements.

Although the motor imagery method provides a good option for the BCI applications, there is a possibility that individuals who have been paralyzed or are amputated for a number of years may not be able to perform motor imagery mental tasks very well [27, 28].

Several researchers have used other non-motor imagery task BCI based on mental task imaginations. They used six EEG channels with electrodes placed on the scalp positions central (C3, C4), parietal (P3, P4) and occipital (O1, O2). The mental tasks chosen included baseline, multiplication, letter composing, figure 3-D rotation and counting [5, 27]. For a practical system, the number of channels needs to be reduced. For example some researchers have used different combinations two and three channels [5, 29]. Our previous study [5] has shown it is possible to reduce from 6 channels to 2 channels without losing accuracy. As a result, in this study, we are focusing on using only two EEG channels for portability and ease of use in a practical system. Looking from the brain function point of view, it has been found that the parietal lobe shows significant activity during mental arithmetic calculation, language and writing skills especially on the left hemisphere [30–32]. Another study showed mental figure rotation created activity in both left and right parietal lobes [33]. A visual task has been shown to produce activity more on the right occipital area [34]. Therefore parietal and occipital areas provide significant features for BCI purposes. Consequently, this paper develops and provides experiment results for a system using two channel wireless EEG placed on the left parietal (P3) and the right occipital (O2) lobes only.

In addition, it has been known for EEG measurement that there is increased amplitude of alpha band (8–13 Hz) during eyes closed action of between 1 and 5 s. This eyes closed phenomenon can be found in normal person and individuals with disabilities and therefore it is reliable enough to be used as a mind switch to create a hand free control system [35].

This study presents the application of biomedical CPS in the integration of the embedded system with the human neural physical system measured by wireless EEG. A hybrid classification system is proposed using a combination of wireless EEG sensing methods which include BCI based mental task, BCI based SSVEP and eyes closed detection. With the purpose of developing a practical real-time biomedical CPS application and for the rapid development of embedded system and wireless technology, this paper presents a microcontroller based CPS system consisting of a separate head-mounted battery-operated wireless EEG sensor communicating with a main embedded system to provide maximum portability, convenience and cost effectiveness. The developed EEG used in this study is a two channel only wireless EEG with the electrodes placed at the back of the head of parietal and occipital lobes.

For the classification algorithm, the linear and non-linear methods have been explored in the EEG signal classification [36]. As an EEG signal is multi-dimensional, a non-linear method namely artificial neural network (ANN) is investigated. The ANN has been widely used in biomedical applications or other engineering applications particularly for classification algorithms [5, 37–39].

Although many studies have reported the implementation of an EEG-based BCI system, there still many subjects unable to use particular mental strategy of EEG-based BCI, known as BCI illiteracy phenomena [40]. As a result, hybrid EEG-based BCI has been developed using the combination of different mental strategies [41]. Currently, most of the hybrid EEG-based BCIs use the combination of motor imagery with SSVEP [40] and P300 with SSVEP [42–45].

Essentially, this paper discusses the development of the novel hybrid BCI by combining mental task (non-motor imagery), SSVEP and eyes closed tasks that have not been explored in previous studies. The use of mental task (non-motor imagery) here is due to the selective motor imagery task defects in patients with severe disabilities. Also, individuals who have been paralyzed for several years may be unable to perform motor imagery tasks effectively. This proposed technique can provide an alternative solution for subjects who cannot use the other combination of hybrid BCI properly. In addition, compared to other hybrid BCI research that uses several EEG channels, this paper proposes the development of hybrid EEG-based BCI using only two EEG channels.

## Methods

### Prototype hybrid BCI system

**Fig. 1:**
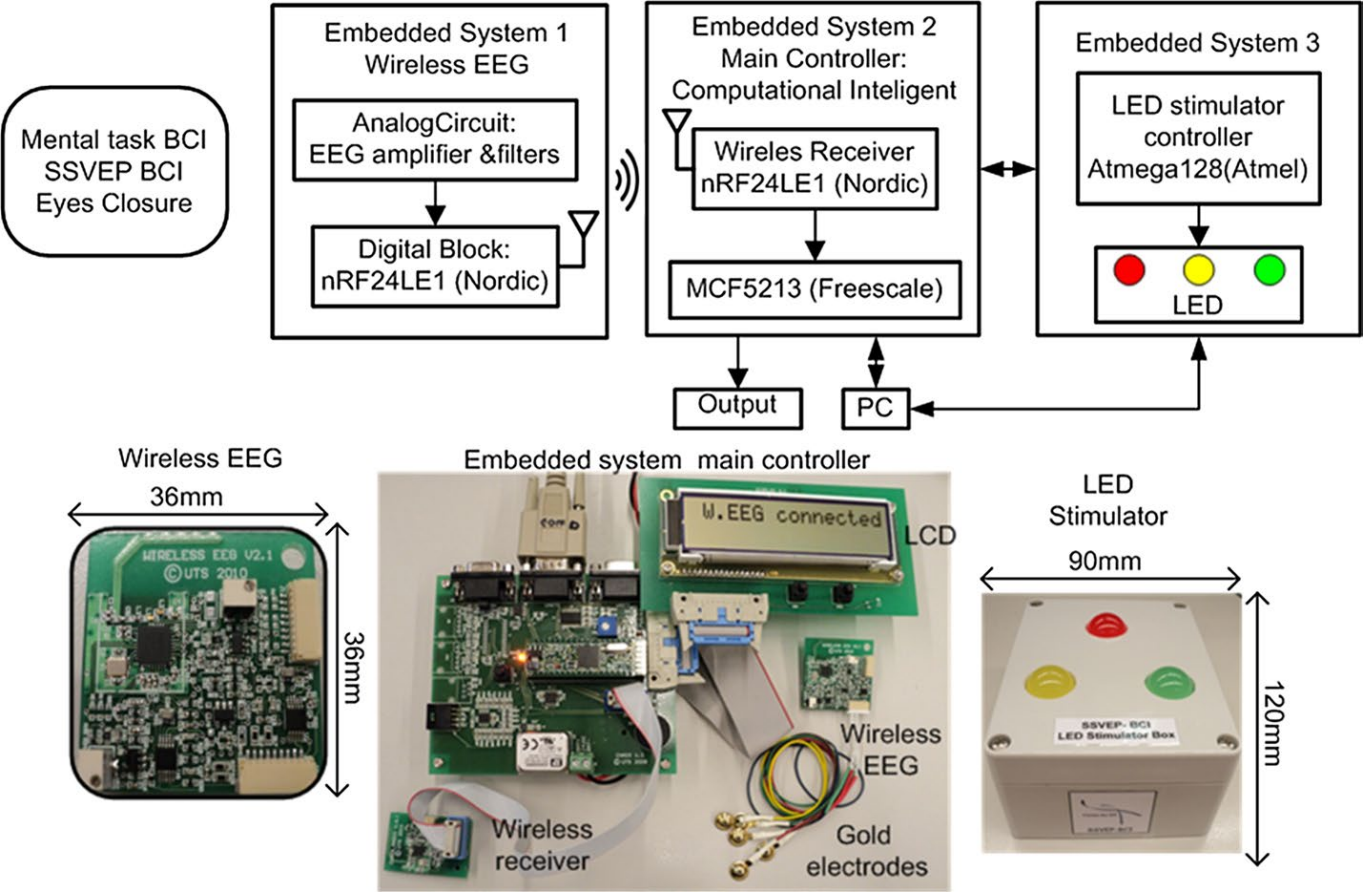
Hybrid BCI system, EEG based CPS module

The proposed hybrid BCI system for biomedical CPS application consists of a combination of mental task based-BCI, SSVEP based-BCI and eyes closed detection using a two channel only wireless EEG and embedded systems. The block diagram in Fig. 1 shows three main modules of the prototype which uses embedded system controllers as functional blocks. The first module is a wireless EEG system which comprises of analog and digital components. The analog section consists of amplifiers and filter circuits. A combined microcontroller and 2.4 GHz RF transceiver, (Nordic Semiconductor) handles the digital section and transfers data wirelessly. The same wireless microcontroller on the receiver captures and sends the data to a second module with the main microcontroller MCF5213 Coldfire^®^ (Freescale™) for signal processing, feature extraction and classification. The third module, an LED stimulator box, consists of the Atmega128 (Atmel^®^) microcontroller to control three LEDs for SSVEP based BCI system.

### Wireless EEG, main controller and stimulator box

The EEG amplifier specification requires a high common mode rejection ratio (CMRR) above 80 dB to tolerate the interfering noise into the system. The amplifier should also be able to detect EEG signals within the range of 5–300 µVolts [46].

**Fig. 2:**
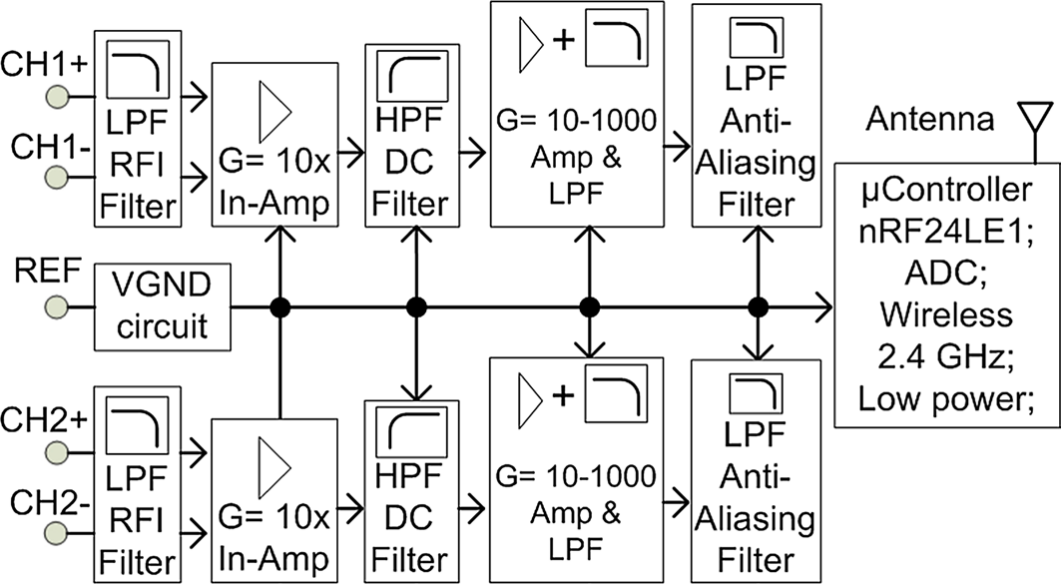
Wireless EEG module

The proposed wireless EEG, as shown in Fig. 2, is divided into analog and digital sections. The EEG is based on two channel bipolar EEG configuration. Each channel has non-inverting inputs (CH1+ and CH2+), inverting inputs (CH1− and CH2−) and a reference input electrode. The amplifier design is based on a DC coupled amplifier which consists of two stages of amplifiers. A precision current mode instrumentation amplifier (In-Amp) AD8553 is used at the first stage which internally contains a voltage to current amplifier, current to voltage amplifier and a high precision auto-zero amplifier. To accommodate the DC offset from the electrodes which could saturate the amplifier, the gain of In-Amp is set at a low value of 10. This is followed by a passive RC high pass filter circuit to remove the DC offset. The second stage amplifier also uses op-amp OPA333 to form a non-inverting amplifier circuit with an adjustable gain up to 1000 using a potentiometer.

To minimize the radio frequency interference (RFI), a differential low pass filter circuit for RF attenuation is attached. An additional filter is provided to the second stage of the amplifier which has an active low pass filter configuration. At the end of the analog block before connection to the microcontroller, an anti-aliasing filter is added. The bandwidth of these filters is 1.5 kHz. The total noise measurement is 3.5 µVolts referring to the input.

The analog to digital converter (ADC) has 12-bit resolution and is configured in differential mode for an improved common mode rejection. For the further noise rejection, four ADC samples are taken and then averaged for each reading. This is particularly effective in removing internally generated noise by the microcontroller. The ADC is configured in differential mode for improved common mode with the least-significant bit of the ADC calculated as follows:

1 $$LSB = \frac{FS}{{2^{n} }}$$

where LSB is least- significant bit, FS is full scale analog input, *n* is the number of bit resolution of ADC, in this case *n* = 12. The ADC setting has configurations which include internal reference of 1.2 V. In differential mode of ADC, the input range swings from –Vref/2 to +Vref/2 which is equal to −0.6 to +0.6 V, so the total full scale (FS) is to 1.2 Volts. LSB can be calculated by using Eq. (1) which gives a result of 293 µVolts (1.2 V/4096). So theoretically, the total gain was 2000 so as to get the input resolution of 0.15 µV (293 µVolts/2000) for 1 LSB. The actual measurement of total noise as shown in Table 1 for the developed wireless EEG was 3.5 µV. According to [46] the EEG signals are within the range of 5–300 µVolts, so the actual noise measured still meets the requirement for EEG signal measurement.

**Table 1:** Wireless EEG specifications

Specs	Value
Dimension	36 mm × 36 mm
Total channels	2
Total electrodes	5 (2 bipolar channel; 1 Ref channel)
Sampling rate	256 Hz
ADC bits	12 bits
CMRR	~95 dB
Noise	3.5 µVolts
Gain	Adjustable up to 10^4^
Current	5 mA
Battery	CR2032 (190–225 mAh)
Battery life	38–45 h

The nRF24LE1™ microcontroller uses the real time clock (RTC) serves as the main ticking of the 256 Hz sampling rate of the system and provides wake up functionality for power saving mode. Most of the routines are handled by an interrupt service routine to create a real-time data acquisition. When the timer interrupt has elapsed, it activates an ADC interrupt. The raw ADC value is transferred via RF 2.4 GHz after attaching it to the transfer protocol. As soon as the wireless receiver detects any incoming data from the interrupt RF routine and the protocol is matched, data is forwarded to a UART for connection to a PC via RS-232 serial line and simultaneously to a serial peripheral bus (SPI) port connected to the main embedded system. The main embedded system using 32 bit MCF5213 Coldfire^®^ from Freescale™ microcontroller which implements the µC/OS real-time operating system (RTOS) which runs several application tasks simultaneously.

The LED stimulation box has three LEDs that flicker at frequencies 6, 13 and 16 Hz. A microcontroller Atmega128 (Atmel^®^) controls the three frequencies stimulus using three separate interrupt timer routines. In BCI, the good frequency stimulator response is between 5 and 20 Hz [47]. The chosen three frequencies are inside this range, excluding the alpha band (8–12 Hz).

### Computational intelligence

In the main microcontroller, a data pre-processor extracts the previous 1 s window of EEG channel readings every quarter second for processing by the features extraction algorithm. As a result, 1 s window data set with some overlap between data sets is obtained every 0.25 s. An overall 10 s data window is used resulting in 37 overlapping segments. To further improve signal quality, the data sets are then passed through two digital signal processing (DSP) filters: first, a moving average filter of 3 data samples width to smooth the signal then a second order Butterworth band-pass filter with the bandwidth 0.5 Hz to 100 Hz.

A power spectral density (PSD) analysis is applied for the features extraction method for mental task based BCI, SSVEP based BCI and eyes closed task. This is done by squaring a 256 point fast Fourier transform (FFT) to every 1 s width data set and converting into the PSD value in frequency bands of EEG: δ (0–3 Hz), θ (4–7 Hz), α (8–13 Hz) and β (14–30 Hz). The δ rhythm is not used due to the low frequency noise such as noise generated from ocular artifact and the γ rhythm is also discarded. As a result, only 27 PSD value (θ, α, β) are formed in each channel and 54 PSD value for two channels.

**Fig. 3:**
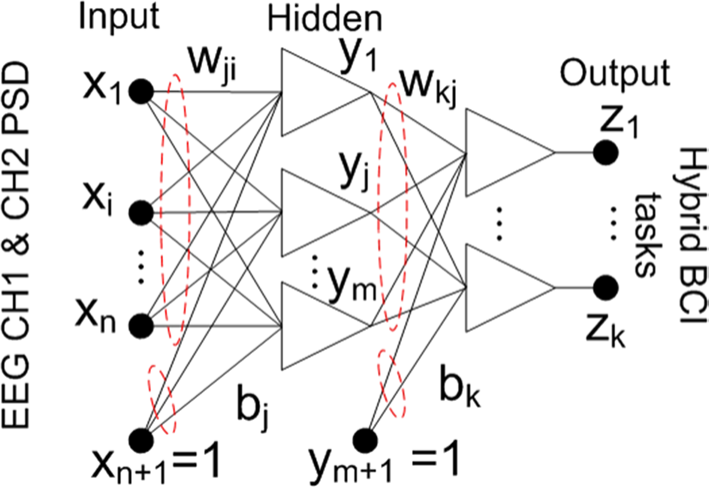
ANN architecture

This study utilizes a multi-layer feed forward back-propagation neural network with one hidden layer network as shown in Fig. 3. A log-sigmoid function was assigned as the activation function which provides data values between one and zero. As a result, prior to the ANN the feature data value needs to be scaled to within the range of zero to one as follows:

2 $$x^{ *} = \left( {x - x_{min} } \right)/\left( {x_{max} - x_{min} } \right)$$

where *x* is the input EEG features (in this case PSD values) before normalization, x* is the input EEG features after normalization, *x*_*min*_ is the minimum value of the input EEG feature and *x*_*max*_ is the maximum value of the input EEG features.

The output vector *z* and the *k*-th component *z*_*k*_ are computed as follows:

3 $$\varvec{z} = \varvec{ }f\left( {{\mathbf{Wx}}^{*} + b} \right)$$

4 $$z_{k} \left( {x,w} \right) = f_{1} \left( {b_{k} + \mathop \sum \limits_{j = 1}^{m} w_{kj} f_{2} \left( {b_{j} + \mathop \sum \limits_{i = 1}^{n} w_{ji} x_{n}^{*} } \right)} \right)$$

where x* represents the input EEG feature vector, or in this case, PSD values from two EEG channels, **W** is the weight matrix vector, *b* is the scalar bias, *z* is the output vector or the three-class classification of hybrid BCI (three mental tasks or three SSVEP tasks), *f*_*1*_ and *f*_*2*_, are the activation functions for each of the two layers used in the ANN and log-sigmoid function used in this paper, *n* is the number of input nodes, *m* is the number of hidden nodes, *k* is the number of output nodes, *w*_*ji*_ is the weight to the hidden unit *y*_*j*_ from input unit *x*_*i*_, *w*_*kj*_ represents the weight to output unit *z*_*k*_ from hidden unit *y*_*j*_. The biases are represented by *b*_*j*_ and *b*_*k*_ respectively.

To accelerate the convergence of the error back propagation learning method, the ANN is trained by the Levenberg–Marquardt algorithm. The data is divided into three datasets: training, validation and testing set. The validation set is used as an early stopping method to make sure the ANN does not over-train.

For the ANN training and classification, the total dataset for mental tasks, SSVEP tasks, eyes closed action for each task per subject is 222 units (37 overlapping segments × 6 sessions), which is divided into training, validation and testing sets of 74 units each. The number of hidden neurons is varied between 4 and 20 neurons until the best classification was obtained with the minimum mean square error (MSE). A validation set is used for ANN training early stop determination.

One of the error cycles performance is shown in Fig. 10. Note that the error (MSE) of the training set decreased smoothly. The validation set dropped from the beginning up to 8 epochs, then started to increase continuously. The training of the network was stopped at 8 epochs because the validation performance started to increase continuously. This is used to prevent over-training of the network. For the performance measurement, classification accuracy for multi-class classification is used in this paper as evaluation criteria as follows:

5 $$Accuracy = \frac{TP + TN}{TP + TN + FP + FN}$$

where *TP* (true positive) refers to a true detection of intentional control (IC) of BCI task being correctly classified as IC event, *TN* refer to no control (NC) of BCI task being classified as NC event. *FP* refers to false detected event or a NC of BCI task being classified as an IC event, *FN* is the IC of BCI task being classified as NC event.

### Hybrid system algorithms

**Fig. 4:**
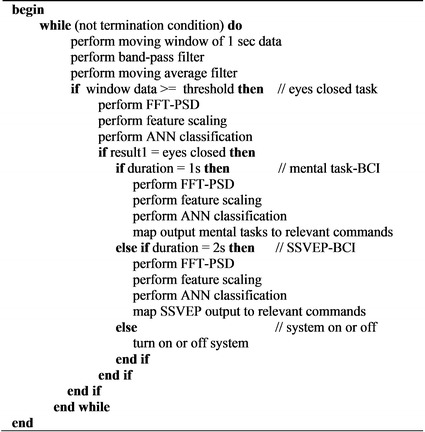
Pseudo-code for hybrid BCI system detection algorithm

The algorithm used for the operation of the hybrid BCI system on the main embedded system controller as shown in Fig. 4. The data packets from the first-in-first-out (FIFO) buffer are passed to the signal processing and classification methods. This is followed by the moving 1 s window data, band-pass Butterworth filter and moving average filter.

**Fig. 5:**
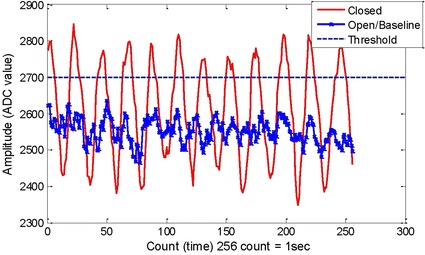
The threshold value of eyes closed signal

At the very first start of the routine as normal condition mode, eyes closed action for a window of 1 s is used to decide whether the BCI control mode is used or not. Here, a window of 1 s data is compared to a threshold for the eyes closed detection. Figure 5 provides the example of the threshold value for one of the subjects used. In this figure the time domain EEG signal of eyes closed swings from 2350 to 2800 of the amplitude or ADC values while the baseline (eyes open) signal has the value between 2400 and 2600 of the ADC value. A safe threshold value at 2700 can be applied for this case so that when the EEG signal is above the threshold, eyes closed signal is detected. If the EEG signal is below the threshold, eyes open is detected.

**Fig. 6:**
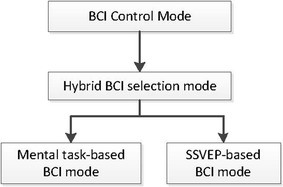
Modes selection in the Hybrid BCI algorithm

After entering the BCI control mode, the eyes closed classification is used again to enter the hybrid BCI selection mode to select different BCI mental strategies. The FFT-PSD algorithm, normalization algorithm and ANN classification are performed to provide the classification of eyes closed task or opened task. The FFT radix-2 of 256 point was written in an assembler language in order to access the special accumulator register for a faster transform operation. The result of eyes closed classification is checked for the duration of the eyes closed action. If the eyes closed is detected for 1 s duration then the system will enter to mental task-based BCI mode which enables the system to classify three mental tasks. In each selection of the BCI type, FFT-PSD, feature scaling/normalization algorithm and ANN is performed to provide the classification of selected BCI. The results of the BCI classification are mapped to the relevant commands. If the eyes closed is detected for 2 s duration then the system will enter to SSVEP-based BCI mode which enables the system to classify three SSVEP frequencies. If the eyes closed is detected for more that 2 s duration, it will turn the system off. Figure 6 shows information of modes for hybrid system algorithm which includes the BCI mode, Hybrid selection mode, mental tasks-based BCI mode and SSVEP-based BCI mode.

## Experimental data collection

### Prototype testing

An initial testing used an EEG simulator (MinSim300 -Netech) to inject a sinusoidal signal with an adjustable frequency and amplitude. The result shows that the wireless EEG has been able to detect the simulation signal with minimum test signal at 10 µV in different frequencies.

The wireless EEG system uses a small square shaped printed circuit board (PCB) with an area of 36 mm^2^ shown in Table 1. There are two EEG channels which include two bipolar montages with five electrodes; two electrodes are used in each channel and one electrode is used for the reference. The experimental CMRR measurement was undertaken by connecting the input of the wireless EEG to a signal generator and the output to the oscilloscope. The signal generator peak to peak amplitude was set to full common mode voltage. The result of CMRR measurement has an average value of about 95 dB at 50 Hz. The gain can be set up to 10^4^. The current consumption of the wireless EEG with the power saving mode programming was 5 mA if the system was operated continuously. The noise measurement was 3.5 µV referring to the input. This current consumption of the wireless EEG is low enough to allow power to be supplied with just a coin cell battery for a battery life of up to 45 h.

**Fig. 7:**
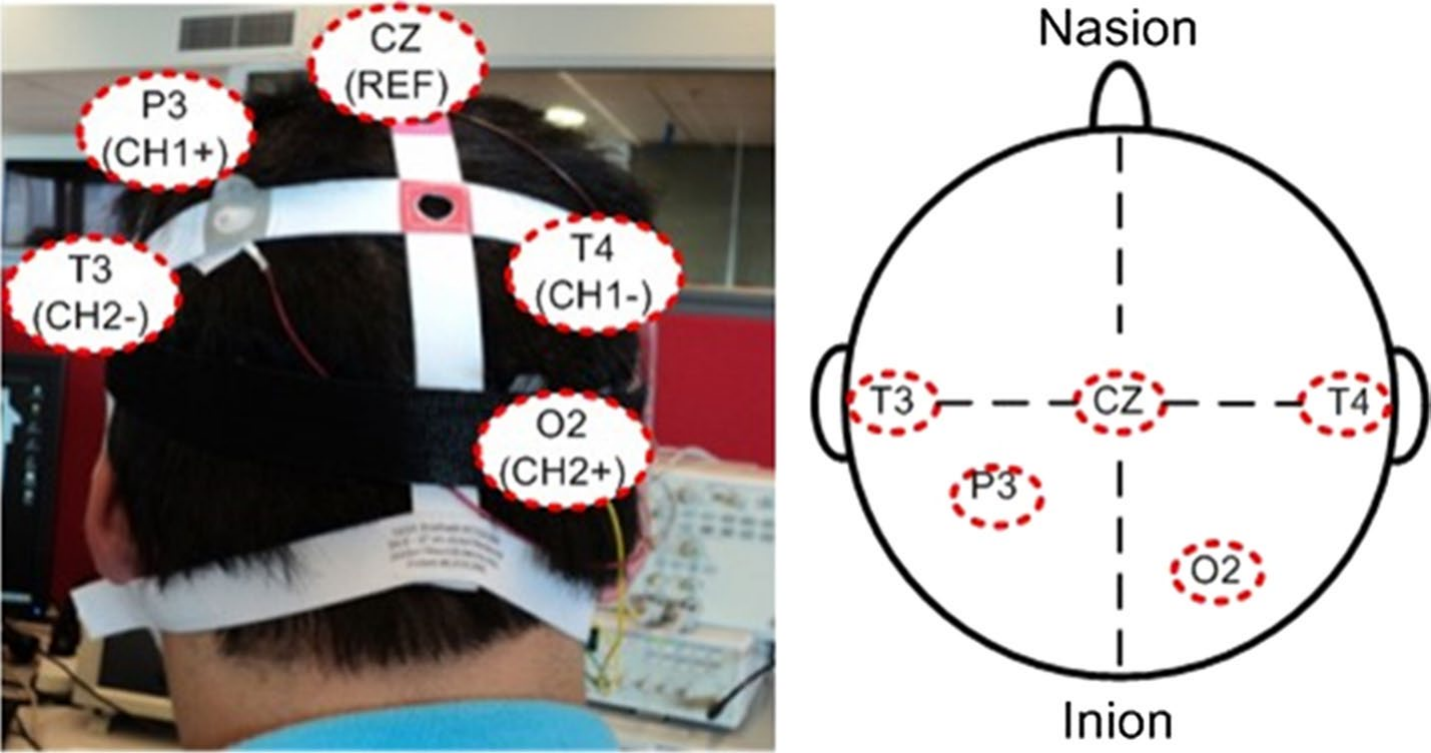
Two channels bipolar EEG electrodes placement

The FFT assembly routine on the MCF5213 microcontroller has an execution time of 520 µs and showed a comparable result with the FFT Matlab function in Fig. 7 with ±3% tolerance.

### Data collection

This study was approved by the Human Research Ethics Committee at the University of Technology, Sydney. EEG signals generated from mental tasks, SSVEP tasks and eyes closed action were collected using the developed BCI prototype from five healthy participants and five patients with high level spinal cord injury (SCI). Healthy participants have ages between 25 and 35 years. The patients with tetraplegia have ages between 45 and 80 years and have levels of cervical SCI impairment at C3, C4, C5 and C6 with the details shown in Table 2.

**Table 2:** Information of healthy subjects and patients with tetraplegia

Participants	Age	Description
S1, S2, S3, S4, S5	25–35	Healthy subjects
SCI1	80	Cervical SCI at level C3 and C4
SCI2	59	Cervical SCI at level C5 and C6
SCI3	50	Cervical SCI at level C3 and C4
SCI4	55	Cervical SCI at level C3 and C4
SCI5	45	Cervical SCI at level C5 and C6

The relevant tasks used are as follows:

- Mental arithmetic: Participants imagined mentally solving a non-trivial multiplication problem.
- Mental figure rotation: Participants were asked to imagine a cube being rolled forward.
- Mental letter composing: Participants composed a simple word in their mind.
- Mental visual counting: Participants performed mentally to visualize appearing and disappearing a number being counting upward on a blackboard.
- SSVEP 6 Hz: Participants were asked to concentrate on an LED that continuously flickers at 6 Hz.
- SSVEP 13 Hz: Participants were asked to concentrate an LED that continuously flickers at 13 Hz.
- SSVEP 16 Hz: Participants were asked to concentrate an LED that continuously flickers at 16 Hz.
- Eyes closed: This task is used to measure alpha wave production. Participants were asked to relax while performing eyes closed action.
- Baseline: Participants were asked to be relaxed with open eyes, thinking of nothing in particular.

The placement of EEG electrodes is based on the international 10–20 system. For each subject gold electrodes were positioned using a bipolar montage at P3-T4 for channel 1 (CH1) and O2-T3 for channel 2 (CH2) as shown in Fig. 7. The location CZ was used for the reference electrode. EEG gel was applied to keep the impedance level low and a better electrical contact. Unnecessary movements and eye blinks were kept as minimal as possible during data collection in each session. Each task was measured for a total of 6 sessions with each session lasting 13 s. The first 3 s of data were discarded for preparation time, the remaining 10 s were used for the further signal processing.

## Results

**Fig. 8:**
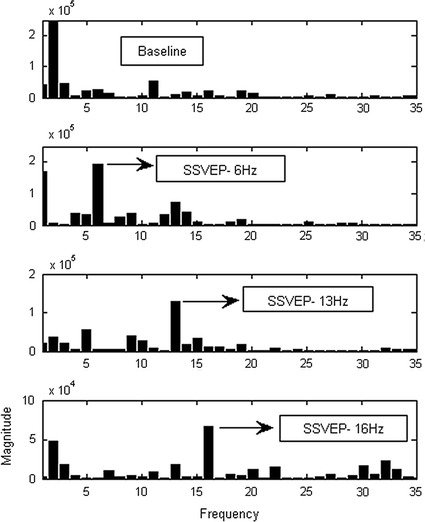
SSVEP-BCI features plotting

There are distinct differences of the data features in terms of amplitude and frequency between SSVEP based BCI and eyes closed action. The SSVEP BCI in the experiment uses three stimulation frequencies: 6, 13 and 16 Hz respectively. Figure 8 shows the plotting of the PSD feature for the three frequencies in which the relevant target frequency can be easily identified from both channels at the highest peak in the power spectrum.

**Fig. 9:**
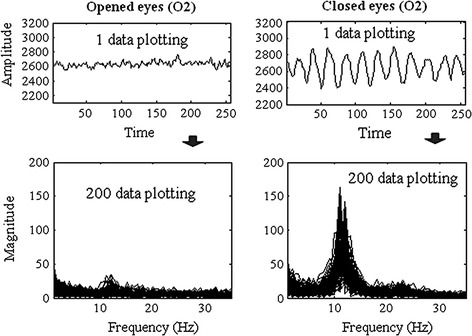
Alpha eyes closed detection with wireless EEG

In practice, the peak frequency during eyes closed action can be used to check and test instrumentation wireless EEG. If the EEG electrodes are properly attached to the scalp, the system should be able to detect the peak alpha frequency during eyes closed action. The time plots in Fig. 9 show a larger amplitude signal from eyes closed compared to eyes opened ones. The dominant alpha frequency between 8 and 13 Hz during eyes closed action on the occipital lobe (O2) also clearly showed in the frequency plots.

The training of the classifier has been developed offline with the training, validation and testing sets taken from five healthy participants and five patients with tetraplegia. Due to the large differences in EEG based-BCI known as inter-subject variability [48] which could affect the performance, in this study, the training of the ANN was done offline with all subjects contributed portions of data for training and testing datasets. In the case of adaptive learning for new user, the system can collect a small sample of data from a new user and re-training the system to enable the classifier to adapt to the data generalization from the new user which was previously unseen by the classifier. This new user adaptive BCI approach will be our future study.

**Fig. 10:**
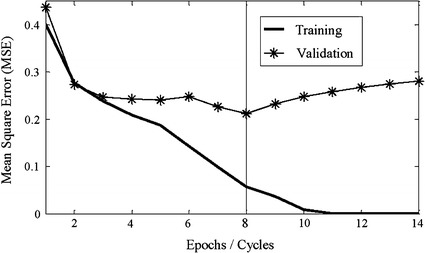
Training and validation error cycle

One of the error cycles performance of the ANN training is shown in Fig. 10. Note that the error (MSE) of the training set decreased smoothly. The validation set dropped from the beginning up to 8 epochs, then started to increase continuously. The training of the network was stopped at 8 epochs because the validation performance started to increase continuously. This is used to prevent over-training of the network.

The results of mental task classification for five healthy subjects are shown in Table 3 with different classification accuracy result for each subject. Table 3 shows each combination of two mental tasks with an accuracy of above 80%. Moreover, the combination of three mental tasks for most of the subjects has accuracy of 72 ± 1%. Additional four mental tasks classification is also shown with the result of 64%. The result of the SSVEP task combination shows a higher classification accuracy compared to the mental task. The average classification on each frequency (6, 13, and 16 Hz) versus baseline task resulted in accuracy of 85 ± 4%, while three frequencies combined classification resulted in accuracy of 80%. The eye closed versus baseline task has classification accuracy of 97%.

**Table 3:** Results of classification accuracy for five healthy subjects (S1-S5)

Task combination	Accuracy (%) of 5 healthy subjects (S1-S5)					
	S1	S2	S3	S4	S5	Mean
Mental tasks						
Arithmetic and rotation	85	83	79	77	85	82
Arithmetic and letter	85	83	86	77	75	81
Arithmetic and count	77	83	90	89	90	86
Rotation and letter	86	91	84	77	78	83
Rotation and count	81	82	82	92	75	82
Letter and count	77	91	90	81	86	85
Arithmetic, rotation and letter	74	75	70	66	69	71
Arithmetic, rotation and count	70	71	73	75	72	72
Arithmetic, letter and count	68	76	79	70	71	73
Rotation, letter and count	69	77	72	71	67	71
Arithmetic, rotation, letter and count	64	67	68	62	62	64
SSVEP tasks						
Baseline and 6 Hz	87	81	85	80	89	84
Baseline and 13 Hz	93	92	86	82	93	89
Baseline and 16 Hz	92	82	90	84	88	87
6, 13 and 16 Hz	83	81	82	78	77	80
Eyes closed tasks						
Baseline and eyes closed	99	92	98	99	98	97

The classification results for patients with tetraplegia are shown in Table 4. The results show comparable classification accuracies to the healthy group. The two mental task classifications still have accuracy of 80% and above. The three mental task classifications yield accuracies of 70 ± 2%, almost the same as in healthy group. The four mental task classifications have accuracy of 62% which is also comparable to the healthy group. The accuracies for SSVEP (6, 13, and 16 Hz) versus baseline task have value between 82 and 84%. The three frequency classifications yield accuracy of 71%. The eye closed versus baseline task classification accuracy is still remaining high of 93%.

**Table 4:** Accuracies for five patients with tetraplegia (SCI1–SCI5)

Task combination	Accuracy (%) of 5 patients (SCI1-SCI5)					
	SCI1	SCI2	SCI3	SCI4	SCI5	Mean
Mental tasks						
Arithmetic and rotation	84	76	87	85	87	84
Arithmetic and letter	88	77	84	77	88	83
Arithmetic and count	89	78	94	94	85	88
Rotation and letter	82	77	76	79	75	78
Rotation and count	89	73	82	82	78	81
Letter and count	80	80	81	91	77	82
Arithmetic, rotation and letter	74	64	67	68	70	68
Arithmetic, rotation and count	79	61	74	76	69	72
Arithmetic, letter and count	75	63	76	75	71	72
Rotation, letter and count	72	64	66	72	62	67
Arithmetic, rotation, letter and count	67	53	63	64	60	61
SSVEP tasks						
Baseline and 6 Hz	80	88	80	76	89	83
Baseline and 13 Hz	90	84	83	83	84	85
Baseline and 16 Hz	89	82.	78	77	90	83
6, 13 and 16 Hz	78	66	70	70	71	71
Eyes closed tasks						
Baseline and eyes closed	96	95	87	88	98	93

Due to the limitation of the recruitment for this study, in which 5 healthy participants (25–35 years old) and 5 patients with tetraplegia (45–80 years old) were available for the experiment. However, the comparison between the healthy group and the patient group on Table 5 shows in relation for combination three mental tasks classification resulted in *p* value >0.2. This means that there were no significant difference between the 5 healthy subjects and the 5 patients with tetraplegia.

**Table 5:** Comparison of healthy subjects and patients with tetraplegia to classify three mental tasks combinations

Three-classes of mental Task Combinations	5 Healthy subjects (mean ± SD)	5 Patients with tetraplegia (mean ± SD)	T-test (*p* value)
Arithmetic, rotation and letter	70.8 ± 3.7	68.6 ± 3.7	0.402
Arithmetic, rotation and count	72.2 ± 1.9	71.8 ± 7.0	0.903
Arithmetic, letter and count	72.8 ± 4.5	72.0 ± 5.4	0.832
Rotation, letter and count	71.2 ± 3.8	67.2 ± 4.6	0.230

## Discussions

It should be noted that the baseline task could sometimes reaches higher amplitude and smaller peak in the alpha bands (8–12 Hz) especially when the subjects are relaxed. Moreover, there is also a dominant alpha wave associated with the eyes closed task. This is the reason why the SSVEP frequencies are chosen not in the range of the alpha band frequency to ensure there is no overlapping during relaxation and during eyes closed action.

The idea of the proposed hybrid system is to combine the mental tasks based BCI, SSVEP based BCI and eyes closed task together with the measurement system just using only a wireless two channel EEG with electrode positions at parietal (P3) and occipital (O2) lobes. The three mental tasks combination is the chosen for BCI applications, for example in wheelchair navigation using three commands (forward, left and right). The SSVEP based BCI with three frequencies combination could be applied to the other applications such as BCI speller system, motor prostheses, environmental control and any other task in the scenario without the subject concentrating on two tasks at the same time. The system is turned on or off by using the eyes closed task with duration of above 2 s. When the system is turned off, the user could perform normal mental activities naturally without the worry of false activation. Eyes closed task with different duration periods is used as well to select between a mental task and SSVEP type of BCI.

Hybrid BCI algorithm (Fig. 4) is applied by using the combination of eyes closed task, mental task based BCI and SSVEP based BCI together with the offline classification result is shown in Table 6 for using ANN classifier. The average ANN classification accuracy of combinations of eyes closed, three mental tasks and three frequencies SSVEP is 74%.

**Table 6:** Results of accuracy of patients with tetraplegia (SCI1-SCI5) with chosen task combinations for hybrid BCI algorithm

Task combination	SCI1–SCI5 = five patients with tetraplegia A1–A5 = accuracies (%); B1–B5 = bit rates (bit/min)											
	SCI1		SCI2		SCI3		SCI4		SCI5		Mean accuracy %	Mean bit rate bit/min
	A1	B1	A2	B2	A3	B3	A4	B4	A5	B5		
Baseline and eyes closed	96	45	95	44	87	26	88	29	98	51	93	39
Arithmetic, rotation and letter	74	30	64	17	67	20	68	22	70	24	69	22
Arithmetic, rotation and count	79	37	61	13	74	30	76	33	69	23	72	27
Arithmetic, letter and count	75	31	63	16	76	32	75	32	71	25	72	27
Rotation, letter and count	72	27	64	17	66	19	72	27	62	14	67	21
SSVEP: 6, 13 and 16 Hz	78	36	66	19	70	24	70	24	71	25	71	25
Mean	79	34	69	21	73	25	75	28	73	27	*74*	*27*

Mean classification for all task combinations = 74% and mean bit rate= 27 bit/min

Another performance measurement technique could also be used, the information transfer rate (ITR) or bit rate as the amount of reliable information received by this hybrid system using the following bit rate function:

6 $$B = V \left[ {\log_{2} N + P \log_{2} P + \left( {1 - P} \right)\log_{2} \left( {\left( {1 - P} \right)/\left( {N - 1} \right)} \right)} \right]$$

where *B* is bit rate (bits/min), *V* is the application speed in trials/mins. *N* is the number of possible tasks and *P* is to the classification accuracy [49]. In general, the ITR depends on the number of classification tasks used, the time of the system for the classification and the classification accuracy. The ITR values previously reported for BCI systems have reached at least 25 bits/min [50].

**Fig. 11:**
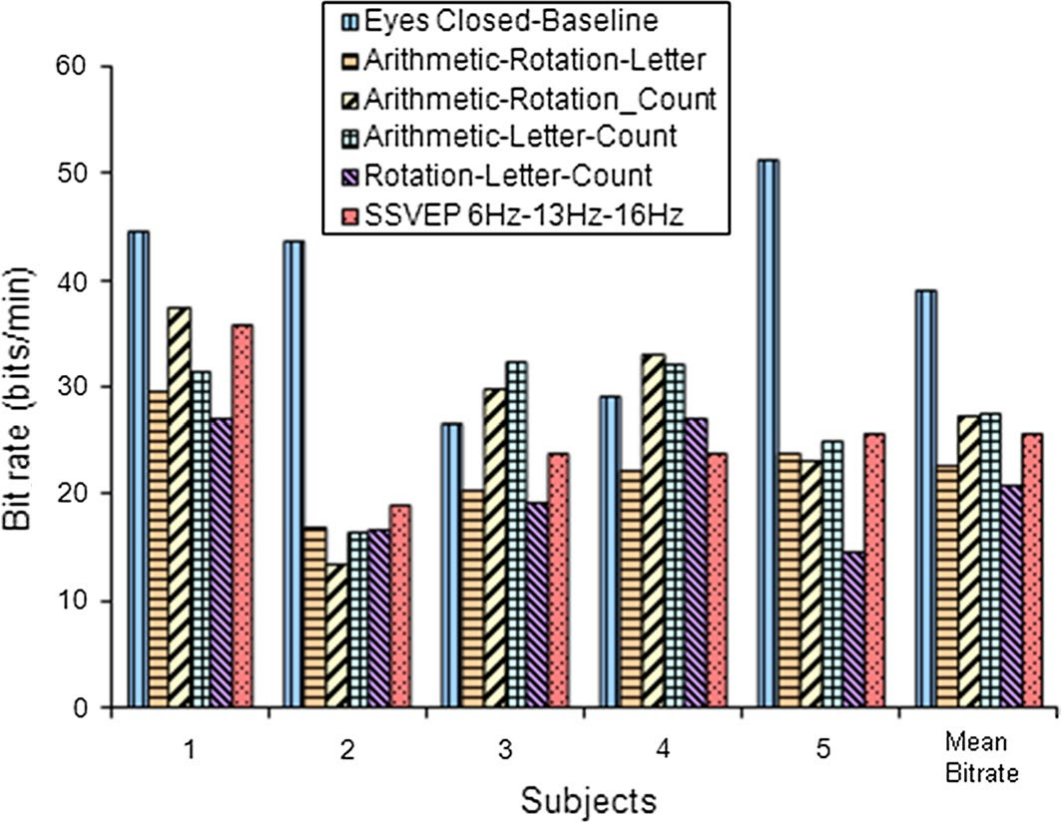
Plotting information transfer rate (ITR) or bit rate in bits/min

In this experiment the system speed is set as variable which could be adjusted. For example Fig. 11 shows the ITR in bits per trial with the speed of the system configured to provide a classification every second (*V* = 60 trials/min). In this prototype experiment, the mean value of the bit rate is between 21 bits/min until 39 bits/min or 27 bits/min in average value for five patients with tetraplegia.

For comparison purposes, the classification accuracy using other classifiers which include linear discriminant analysis (LDA) [51, 52], support vector machine (SVM) [52, 53] as shown in Table 7, together with the result of ANN. The average classification accuracy between the combinations of eyes closed, three mental tasks and three frequencies shows that the LDA classifier gives a lower average accuracy of 63.7% compared to SVM and ANN. The SVM classifier is comparable to ANN with accuracy of 73.2%. The ANN classifier with the average accuracy of 74% is the algorithm being implemented in the embedded system for real-time classification.

**Table 7:** Comparison accuracy of the classifiers (LDA, SVM and ANN) of patients with tetraplegia (SCI1-SCI5) with chosen task combinations for hybrid BCI

Tasks combination	Mean of accuracy from five patient with tetraplegia		
	LDA (%)	SVM (%)	ANN (%)
Baseline and eyes closed	80	91	93
Arithmetic, rotation and letter	60	70	69
Arithmetic, rotation and count	61	71	72
Arithmetic, letter and count	61	70	72
Rotation, letter and count	60	66	67
SSVEP: 6, 13 and 16 Hz	60	72	71
Average for all tasks	63.7	73.2	*74*

Average accuracy for all tasks of proposed method ANN =74%

**Fig. 12:**
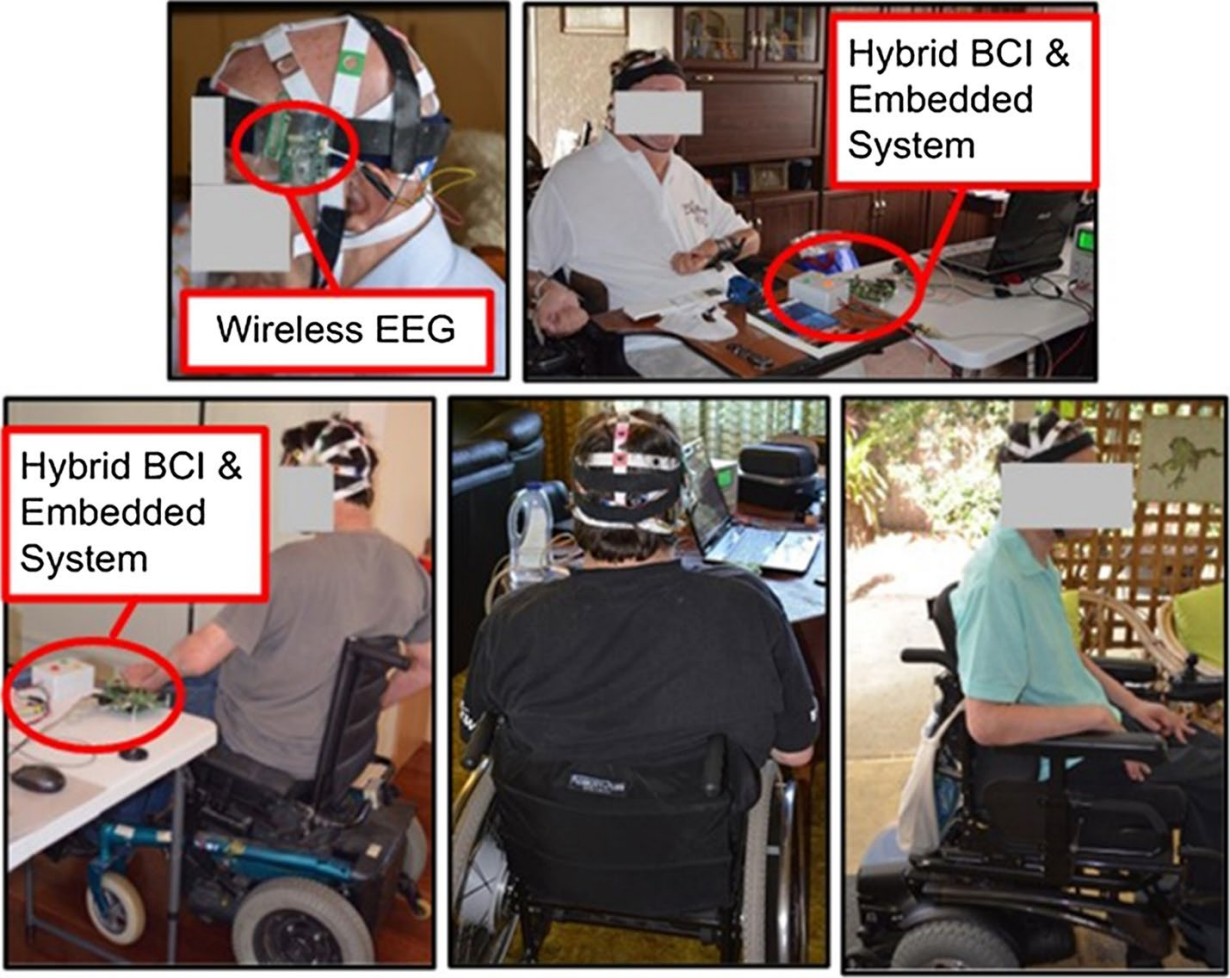
Experiment of hybrid BCI system with patients with tetraplegia

The final weights from ANN training giving the best classification accuracy of the tasks are transferred as parameters to the main embedded system for real-time application. Further testing of the real-time embedded hybrid BCI system has been conducted for patients with tetraplegia to prove that the hybrid task is adequate to be used for the intentional signal to control the wheelchair. The three-task classifications from Table 6 are mapped to the three wheelchair movements (left, forward and right). The eyes-closed task is used to switch the system on/off and for hybrid system selection. The set-up experiment of patients with tetraplegia is shown in Fig. 12.

**Fig. 13:**
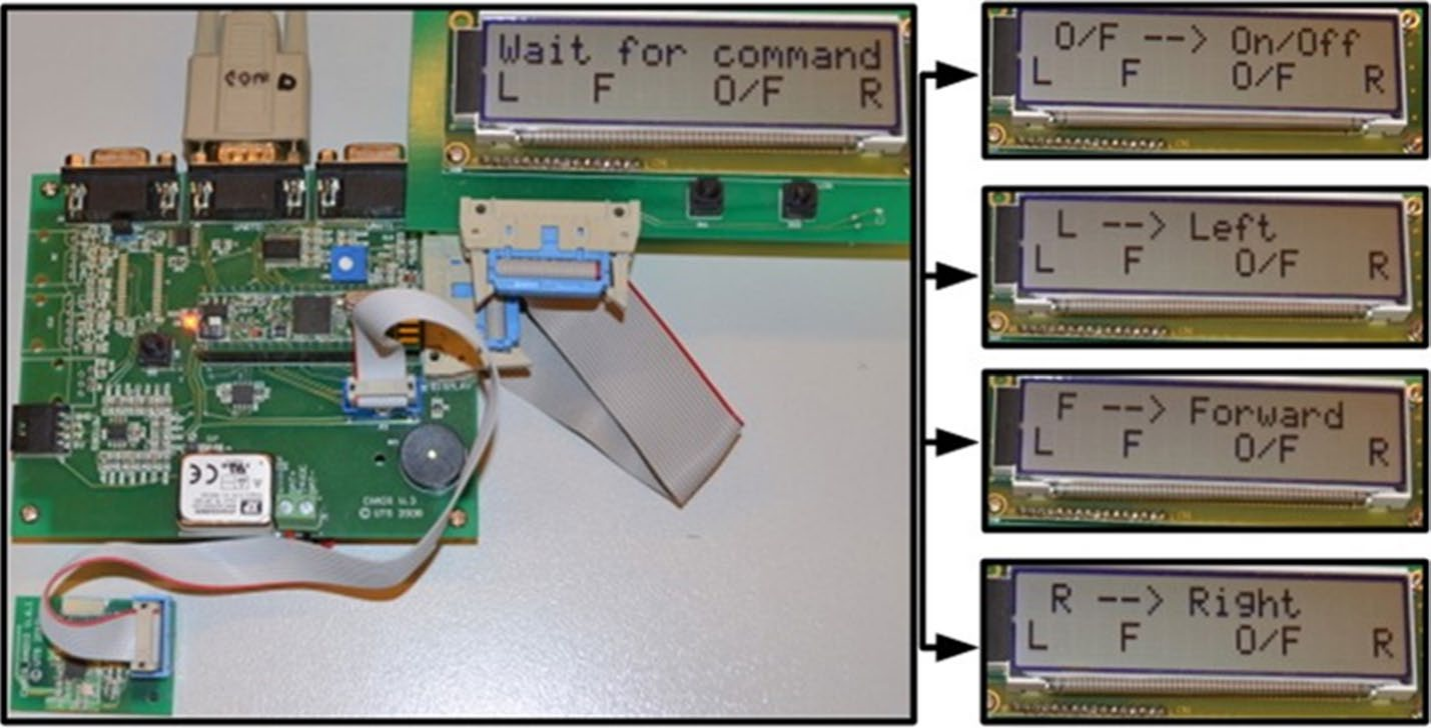
Real-time BCI detection, mapped to commands and LCD output

The LCD displays the output of the command recognition as shown in Fig. 13 which is related to the hybrid BCI tasks. The embedded system turns into a waiting state for any command activation. As soon as a command is activated, the LCD displays the result of the classification on the first line of LCD. If the on/off command is activated, LCD displays ‘O/F → On/Off’. If the turn left command is activated, LCD displays ‘L → Left’. For move forward command, LCD displays ‘F → Forward’ and for turn right command, LCD display ‘R → Right’. The second line of the LCD displays the same information: ‘L F O/F R’ to represent four intentional signals of wheelchair control.

The result of the real-time testing with the five patients with tetraplegia is shown in Table 8. Essentially, there are four basic commands used for the wheelchair which includes: on/off, left, forward and right. Eyes closed task is used to switch the system on and off. The chosen best classification of three mental tasks or three frequencies SSVEP (Table 6) is used to provide three wheelchair steering states: turn left, move forward and turn right. Each particular task related to each command is repeated 25 times for the testing experiment. The correctly detected commands and speed of execution are identified.

**Table 8:** Results of real-time testing for 5 patients with tetraplegia with the repeated command of intentional signals

Five patients with tetraplegia (SCI1 -SCI5)	Testing wheelchair commands eyes closed for on/off command and user chosen 3-task for left, forward, right commands (each runs at 25 times)								% Mean of success rate	Speed of execution (seconds)
	On/Off		Left		Forward		Right			
	Correctly identify1	% Success rate1	Correctly identify2	% Success rate2	Correctly identify3	% Success rate3	Correctly identify4	% Success rate4		
SCI1	24	96	18	72	16	64	17	68	75	2–3
SCI2	20	80	16	64	14	56	15	60	65	2–4
SCI3	20	80	17	68	16	64	16	64	69	2–3
SCI4	21	84	18	72	16	64	17	68	72	2–4
SCI5	22	88	15	60	17	68	16	64	70	2–4
Mean	21	86	17	67	16	63	16	65	*70*	*2*–*4*

The average speed of execution in real-time is 2 to 4s

For the on and off command using eyes closed task, the success rates among five patients with tetraplegia are still high enough at between 80 and 96%. This is relevant to the ANN classification result of the eyes closed task. Testing for the ‘turn left’ command indicates success rates of between 60 and 72%. The success rates detection for the ‘move forward’ and turn right commands are between 60 and 68%. The success rates of these three wheelchair steering movements are relevant to the ANN classification result of the three mental tasks or 3 SSVEP frequency classifications. The average success rate of the detection from five patients with tetraplegia is 70%. The speed of execution each task is varied from 2 to 4 s.

Note that this study used data from 5 healthy participant and 5 patients with tetraplegia. The limitation of current algorithm may not effectively work for new user. In the case of adaptive learning for new user, the system can collect small sample for re-training to enable the generalization of the classifier. This new user adaptive BCI study will be our future study.

## Conclusion

The prototype of hybrid brain computer interface for the biomedical cyber-physical system application has been presented here which has a small size two channel wireless EEG, microcontroller based system and accompanying low power embedded processing system with portability, convenience and cost effectiveness compared to other PC based systems. The hybrid system is successfully able to combine the mental task classification using three mental task combinations, SSVEP based BCI with three frequencies classification and eyes closed detection by using only two EEG channels with active position at parietal (P3) an occipital (O2) lobes. This experiment is involved with five healthy subjects and five patients with high level injury SCI (tetraplegia).

The developed hybrid BCI system provides more flexibility for the user to choose the suitable task. The results show comparable offline classification accuracies between healthy subjects and patients with tetraplegia. For patients with tetraplegia, the average offline ANN classification accuracy of the combination of mental task based-BCI, SSVEP based BCI and eyes closed is 74% and the average information transfer rate is 27 bits/min. For the real-time testing of the intentional signal on patients with tetraplegia, the average success rate of detection is 70% and the speed of detection varies from 2 to 4 s. Future direction of this research is the real-time brain controlled wheelchair and the use of the sensor for obstacle avoidance for the safety to create a semi-autonomous brain controlled wheelchair system.

## Abbreviations

ADC: analog to digital converter
ALS: amyotrophic lateral sclerosis
ANN: artificial neural network
BCI: brain computer interface
CPS: cyber-physical system
CMRR: common mode rejection ratio
EEG: electroencephalography
ERD: event-related desynchronization
ERS: event-related synchronization
FFT: fast Fourier transform
FIFO: first-in-first out
In-Amp: instrumentation amplifier
ITR: information transfer rate
MSE: mean square error
PCB: printed circuit board
PSD: power spectral density
RFI: radio frequency interference
RTC: real time clock
RTOS: real-time operating system
SCI: spinal cord injury
SMR: sensory motor rhythm
SPI: serial peripheral bus
SSVEP: steady state visual evoked potential
UART: universal asynchronous receiver/transmitter
